# Evaluation of Optimal Reference Genes for qRT-PCR Analysis in *Hyphantria cunea* (Drury)

**DOI:** 10.3390/insects13010097

**Published:** 2022-01-14

**Authors:** Xudong Zhao, Yishu Geng, Tianyi Hu, Yongang Zhao, Suling Yang, Dejun Hao

**Affiliations:** 1 Co-Innovation Center for Sustainable Forestry in Southern China, Nanjing Forestry University, Nanjing 210037, China; Zhaoxudong032z@outlook.com (X.Z.); angel520-007@163.com (Y.G.); njfuhu@163.com (T.H.); 2 College of Forestry, Nanjing Forestry University, Nanjing 210037, China; 3 Forest Station of Huaian District, Huaian 223001, China; yongang67@163.com (Y.Z.); yslyslysl73@163.com (S.Y.)

**Keywords:** reference genes, normalization, *Hyphantria cunea* (Drury), qRT-PCR

## Abstract

**Simple Summary:**

Reverse transcription-quantitative polymerase chain reaction (qRT-PCR) has rapidly become the most accessible and widely-applied method for quantifying gene transcription. Stable reference genes need to be normalized to facilitate gene expression studies and obtain more accurate qRT-PCR data. In this research, eight reference genes from different functional categories and gene families were used to assess the stability level of gene expression under different conditions in *H. cunea*. Finally, the expression pattern of the target gene serine proteases (*HcSP1*) was evaluated under different conditions to verify the precision and reliability of the results. This study provides a comprehensive list of suitable reference genes for analyses and optional reference genes for other gene function research in *H. cunea*.

**Abstract:**

The relative quantification of gene expression is mainly achieved through reverse transcription-quantitative PCR (qRT-PCR); however, its reliability and precision rely on proper data normalization using one or more optimal reference genes. *Hyphantria cunea* (Drury) has been an invasive pest of forest trees, ornamental plants, and fruit trees in China for many years. Currently, the molecular physiological role of reference genes in *H. cunea* is unclear, which hinders functional gene study. Therefore, eight common reference genes, *RPS26*, *RPL13*, *UBI*, *AK*, *RPS15*, *EIF4A*, *β-actin*, *α-tub*, were selected to evaluate levels of gene expression stability when subjected to varied experimental conditions, including developmental stage and gender, different tissues, larvae reared on different hosts and different larval density. The geNorm, BestKeeper, ΔCt method, and NormFinder statistical algorithms were used to normalize gene transcription data. Furthermore, the stability/suitability of these candidates was ranked overall by RefFinder. This study provides a comprehensive evaluation of reference genes in *H. cunea* and could help select reference genes for other Lepidoptera species.

## 1. Introduction

The fall webworm, *Hyphantria cunea* (Drury) (Lepidoptera: Noctuidae), is a polyphagous leaf-eating pest. It is native to North America and has now spread to more than 32 countries in Asia and Europe [1,2]. In 1979, it was first reported in China as a significant invasive species on the international quarantine list and it is distributed across 13 provinces in China [3,4,5].

The rapid adaptability of the fall webworm is probably due to its high degree of polyphagy and fecundity [6]. The larvae feed on more than 400 species of deciduous tree leaves, including garden trees in urban areas [7]. In its native areas, their activity does not cause significant damage because there are natural enemies. However, the insect has caused considerable loss to host plants, including the consumption of nearly-whole leaves in newly distributed areas [2].

The ecology and physiology of *H. cunea* have been extensively studied in recent years [4,8,9,10,11,12], but their molecular mechanisms are still unclear. The advent of next-generation sequencing methods makes it possible to investigate the genetic foundation of its physiology and biology.

Due to its high sensitivity, rapidity, specificity, and accuracy [13,14,15], qRT-PCR has been widely used to analyze functional gene transcription and expression levels in animals, plants, and microorganisms [16,17,18,19,20]. However, batch-to-batch variations in RNA extraction and variable efficiency of cDNA synthesis have limited the accuracy and reliability of qRT-PCR [13,21]. Besides, several other factors can also affect the precision and authenticity of qRT-PCR results, such as the accuracy of the biological signals, the yield of the RNA extraction process, quantity and quality of templates used, differences in polymerase reactions, amplification efficiency of primers, and the stability of reference genes [22,23,24]. Therefore, during qRT-PCR assays, it is necessary to normalize the gene transcription data to ensure more accurate and reliable results when comparing the expression of reference and target genes in the same sample after correcting for unnecessary experimental variation.

Recently, ribosomal protein L13 (RPL13), glyceraldehyde-3-phosphate dehydrogenase (GAPDH), ribosomal protein 32 (RP32), actin, β-actin, β-tubulin (β-tub), elongation factors 1a (EF1α) were selected as reference gene(s) to study the function of indicator genes in *H. cunea* [4,8,9,10,11,12,25,26]. However, the stability of reference genes, in abiotic or biotic conditions, has not been systematically evaluated and validated. Therefore, there is an urgent need to identify and validate reference genes suitable for *H. cunea*.

In this study, the expression stability of eight candidate reference genes was assessed in *H. cunea*, including RPL13, arginine kinase (AK), eukaryotic initiation factor 4A (EIF4A), α-tubulin (α-tub), polyubiquitin (UBI), ribosomal protein S26 (RPS26), ribosomal protein S15 (RPS15), and β-actin. These reference genes were selected from different functional categories and gene families to prevent the influence of co-regulation. These candidate genes were assessed under varying abiotic conditions (i.e., different hosts, larval density) and biotic factors (i.e., developmental stage and gender and different tissues) by using the four statistical algorithms: geNorm [27], NormFinder [28], BestKeeper [29], and the ΔCt method [30]. Moreover, RefFinder, a comprehensive platform that can integrate the above-mentioned algorithms, comprehensively compared and ranked the overall stability of these candidates [31]. Our results provide a comprehensive assessment of the reference genes and validate their stability and reliability in *H. cunea*, and they also provide a valuable resource to select reference genes for other lepidopterans.

## 2. Materials and Methods

### 2.1. Insect Culture

Third instar larvae were collected from *Populus deltoides* (Marshall) in Huaian county of Jiangsu Province (33.62° N, 119.02° E) in May 2020, and were reared with fresh *Populus* leaves in a light incubator at 25 ± 1 °C with a 16 h light/8 h dark cycle and 60 ± 5% humidity. Leaves were replaced every two days until the larvae pupated. Pupae were sexed based on the morphology of a few terminal abdominal segments and maintained under the same conditions as larval rearing. A total of 80 newly emerged adults (at a ratio of 1 female to 3 males) were placed in screen cages (40 × 50 × 30 cm) at room temperature for mating and oviposition.

### 2.2. Experimental Treatments

#### 2.2.1. Developmental Stages and Gender

Samples were collected from each developmental stage and gender of *H. cunea* and included 200 eggs, 10–20 1st–6th instar larvae, 5 pupae (3 days old), 5 male and female adults (2 days old). Then, they were flash-frozen in liquid nitrogen and stored at −80 °C until use. Each sample was collected in triplicate.

#### 2.2.2. Tissues

Tissue samples (head, silk gland, midgut, cuticle, Malpighian tubule, and fat body) were collected from 10 4th instars (2 or 3 days-old) following molting, which were dissected under a binocular microscope by using a sterilized scalpel and tweezers on ice. Each sample was treated and stored as above.

#### 2.2.3. Reared on Different Hosts

The newly-hatched larvae were obtained from an established population reared on artificial diets, classified into four groups, and maintained on different host plants for one generation. The 4th instar larvae from the next generation were used for collection. The four plant species examined were *Populus deltoides* (Marshall), *Camptotheca acuminata*, *Cerasus serrulata* var. lannesiana, *Cerasus serrulata* (Lindl.). Each sample was treated and stored as above.

#### 2.2.4. Different Larval Density

The fall webworm colony was reared for two generations on artificial diets. In the first-generation, the larvae were reared at a fixed density (50 larvae/box (21.5 × 14.5 × 7 cm)). Subsequently, in the next generation, newborn larvae were classified into six density groups of 1, 3, 6, 9, 12, and 15 larvae per Petri dish (diameter 8 cm). For all the density treatments, the number of larvae was kept the same during the feeding period. New artificial diets were supplemented every day. Each sample was treated and stored as above.

### 2.3. Total RNA Isolation and cDNA Synthesis

According to the manufacturer’s protocol, total RNA was isolated from each sample using TRIzol reagent (Invitrogen, Carlsbad, CA, USA), and subsequently treated with DNase-treatment (Vazyme, Nanjing, China). The RNA integrity and purity were determined by 1% agarose gel electrophoresis and assessed by a Nano-Drop 2000 Spectrophotometer (Thermo Scientific, Wilmington, DE, USA). Finally, 1000 ng of total RNA were used to synthesize cDNA by HiScript 1st Strand cDNA Synthesis Kit (Vazyme, Nanjing, China), then applied to both PCR and qRT-PCR.

### 2.4. Identification of Candidate Reference Genes, One Target Gene, and Primers Design

Based on *H. cunea* transcriptomic data (unpublished), sequences corresponding to eight candidate reference genes (*β-actin*, *RPL13*, *AK*, *EIF4A*, *α-tub*, *UBI*, *RPS26*, and *RPS15*) were amplified by PCR from *H. cunea* cDNA. The primer sequences used for gene cloning are shown in the Supplementary Materials, Table S1. The PCR program included 3 min at 95 °C, 30 cycles of 95 °C for 10 s, 50 °C for 15 s, and 72 °C for 50 s, and 72 °C for 5 min. The PCR products were detected by 1.5% agarose gel and extracted by a DNA purification kit (TIANGEN, Beijing, China). Subsequently, the fragments were ligated to pCE2 vector and transformed into *DH5α* competent cells (Vazyme, Nanjing, China). Afterward, the positive clones were selected randomly for each construct and sequenced by SHENGGONG BIOLOGY (Shanghai, China). After verifying the candidate reference genes, an online tool (http://www.primer3plus.com/cgi-bin/dev/primer3plus.cgi#opennewwindow 22 November 2021) was used to design the primers for qRT-PCR analyses. The specificity of primers was determined based on qRT-PCR and melting curve analysis. The presence of individual peaks confirmed the specificity of amplicons.

### 2.5. qRT-PCR

The qRT-PCR reactions were performed in the ABI 7500 System (Applied Biosystems, Foster City, CA, USA). A total of 20 µL reaction volume was configured by the protocol of Hieff UNICON^®^ qPCR SYBR Green Master Mix (YEASEN, Shanghai, China), and contained 10 µL 2 × SYBR Premix Ex Taq™ II, 0.4 µL (10 μM) of each gene-specific primer, 1.2 µL of cDNA, and 8 µL of ddH_2_O. The thermal cycling conditions were as follows: 95 °C for 5 min, 40 cycles of 95 °C for 10 s, and 60 °C for 40 s. Subsequently, a melting curve analysis was conducted in the 60–95 °C temperature range to verify the consistency and specificity of each reaction product.

A series of 10-fold dilutions of cDNAs from 500 ng/µL to 0.05 ng/µL were used to make the five-point standard curves by using a linear regression model. The following equation was used to estimate the qRT-PCR amplification efficiency (E) of all genes: E = (10^[−1/slope]^ − 1) × 100%.

### 2.6. Expression Stability of Candidate Reference Genes under Different Conditions

The ABI 7500 System software (ver. 2.3) (Applied Biosystems, USA) was used to analyze the qRT-PCR data. In this study, the expression stability of each candidate gene was calculated and ranked by four statistical algorithms: geNorm [27], BestKeeper [29], NormFinder [28], and the comparative delta Ct algorithm [30].

The GeNorm algorithm appraised the stability of the candidate reference gene on the basis of the “M” value [27]. The lowest M value indicated the most stable expression genes. Moreover, GeNorm also determined the optimal number of reference genes. The pairwise variation value calculated by geNorm (Vn/Vn+1) was below 0.15, suggesting no additional reference genes were required for normalization. NormFinder calculated the stability value (SV) of candidate reference genes. The lower the SV, the more stable they were [28]. The BestKeeper program was used to evaluate standard deviations (SD) of the Ct values, which determined the stability of reference genes [29]. The comparative ΔCt method calculated the mean standard deviation of Ct values for candidate reference genes and then determined the stability based on the delta Ct values. Candidate reference genes with higher delta Ct variability were considered as the least stable.

Finally, a web-based comprehensive tool, RefFinder (https://www.heartcure.com.au/reffinder/ 10 December 2021), which can integrate the four computational programs (geNorm, BestKeeper, Normfinder, and the ΔCt methods), was used to comprehensively compare and rank the candidate reference genes [31]. It assigned an appropriate weight to each gene and calculated the geometrical mean of their weights for the final rank.

### 2.7. Validation of Reference Genes

To validate the recommended reference genes, we selected the serine proteases (*HcSP1*) that are widely distributed in insects as the target gene. They are the most dominant digestive enzymes and play an important role in the protein hydrolysis process in Lepidopteran larval guts, accounting for about 95% of the digestive activity [32,33]. The transcription levels of *HcSP1* were estimated in different development stages, tissues, larval density, and fourth instar larvae after feeding on different host plants. qRT-PCR amplification of *HcSP1* (Accession number: MH663425) was performed with primers as follows: Forward (AACAGGAATAACCGCCCACTAC) and Reverse (GGCAAGGATCCAGCTAATGAAA). The relative expression levels of *HcSP1* were determined according to the 2^−ΔΔCt^ method [34].

## 3. Results

### 3.1. Amplification Specificity and Efficiency of Candidate Reference Genes

Agarose gel electrophoresis and melting curve analysis by qRT-PCR were performed to evaluate the amplification specificity and efficiency of the primers. The results indicated that all the primers of candidate genes showed a single amplicon with a predicted strip size ranging from 92 to 165 bp on 1% agarose gel (Figure S1A). Furthermore, melting curve analysis exhibited individual peaks that confirmed the amplification specificity of primer pairs (Figure S1B), and all amplicons were sequenced and showed 99–100% similarity with the transcriptomic sequences. The efficiency (E) of qRT-PCR and correlation coefficient (R^2^) for each standard curve are shown in Table S1. The efficiencies of all tested primer pairs ranged from 87.0 for *HcSP1* to 127.4% for *RPL13*, with associated R^2^ values of 0.974–0.999 (Table S1).

### 3.2. Expression Profiles of Candidate Reference Genes

**Figure 1:**
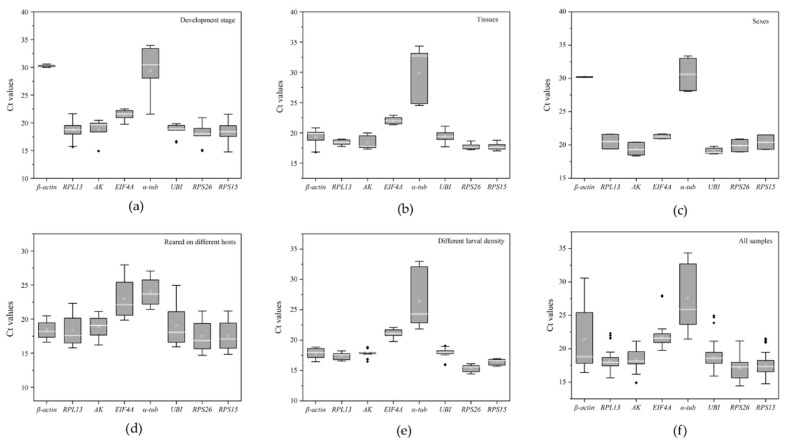
Box-whisker plots of expression patterns of candidate reference genes under different conditions. (**a**) Developmental stages. (**b**) Different tissues. (**c**) Gender. (**d**) Larvae feeding on different hosts. (**e**) Different larval densities. (**f**) All samples. The white line denotes the median in the box. The interquartile range is denoted by the upper and lower edges, which represent the 75th and 25th percentiles. The whisker caps on each box represent the maximum and minimum values.

The cycle threshold (Ct) values under different conditions were calculated by qRT-PCR to evaluate the transcription levels of the eight candidate reference genes (Figure 1). The Ct mean values ranged from 14.4–34.3 cycles. *α-tub* exhibited a mean Ct-value > 26 cycles, while the other candidate reference genes (*β-actin*, *RPL13*, *AK*, *EIF4A*, *UBI*, *RPS26*, and *RPS15*) ranged from 14 to 22 cycles (Figure 1a–f). *RPS26* (average Ct value = 17.08) and *RPS15* (average Ct value = 17.50) were expressed at the highest levels while *α-tub* (average Ct value = 27.61) was expressed at the lowest level. Moreover, the gene *β-actin* showed the greatest variation (SD ± 4.42) in the Ct values (ranging from 16.45 to 30.57), while *RPL13* showed the smallest variation (SD ± 1.08) in Ct values (ranging from 15.63 to 22.30) (Figure 1f).

### 3.3. Expression Stability of Candidate Reference Genes under Different Conditions

The results of the analysis of the stability of the candidate reference genes were as follows.

#### 3.3.1. Developmental Stage and Gender

**Figure 2:**
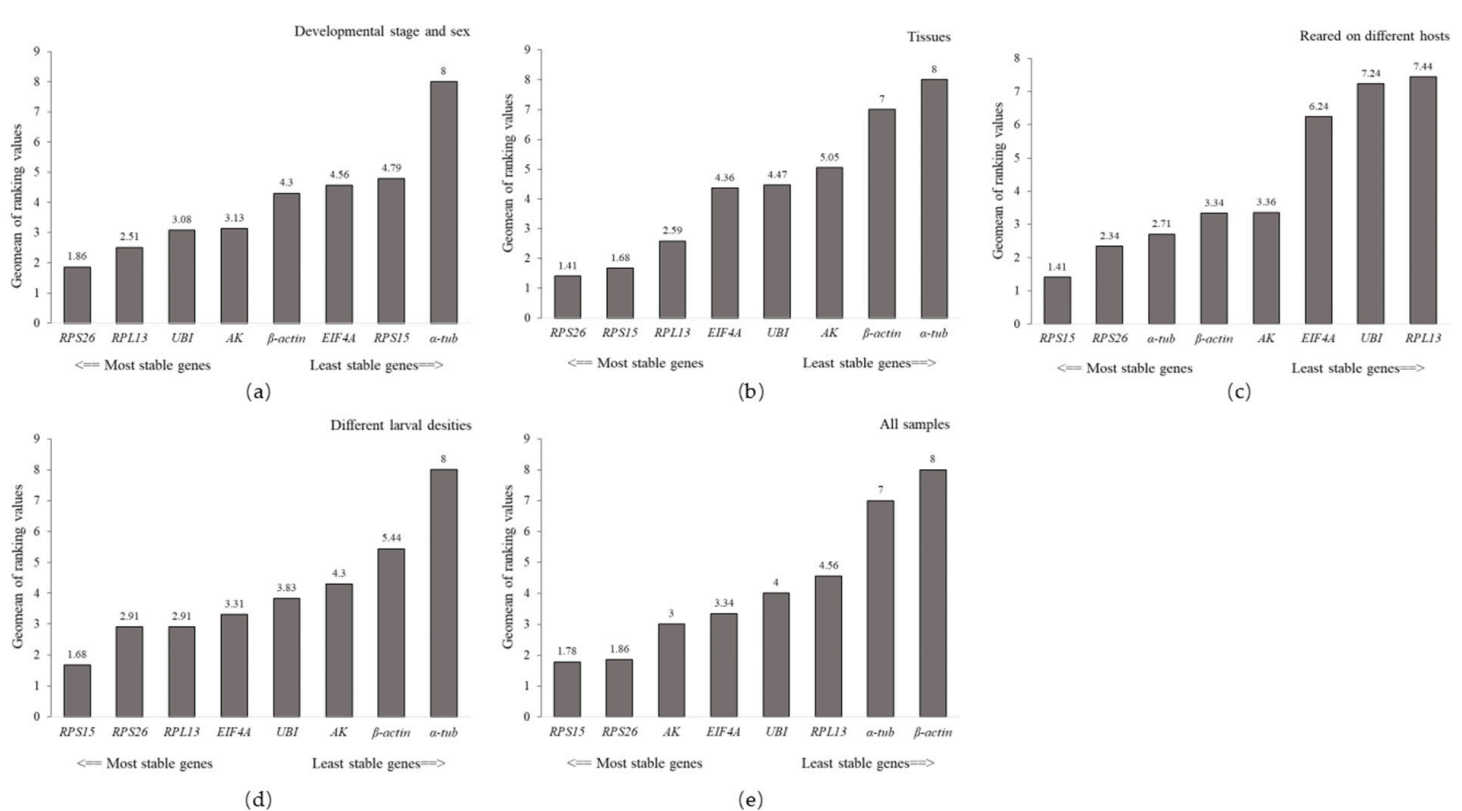
The stability of the eight candidate reference genes was evaluated by the RefFinder. (**a**) Developmental stages and gender. (**b**) Different tissues. (**c**) Larva feeding on different hosts. (**d**) Different larval densities. (**e**) All samples.

The BestKeeper and NormFinder indicated that *β-actin* and *AK* were the most stable in different developmental stages and gender, respectively. In contrast, *α-tub* exhibited the greatest variation (Table 1). Based on the ΔCt method, *RPS26* and *RPL13* were the two most stable reference genes (Table 1). Similarly, the lowest M (0.205) value for the *RPS26*/*RPL13* pair was calculated by GeNorm, suggesting they were the most stable transcripts. The RefFinder ranked the stability of candidate genes from highest to lowest as follows: *RPS26* > *RPL13* > *UBI* > *AK* > *β-actin* > *EIF4A* > *RPS15* > *α-tub* (Figure 2).

#### 3.3.2. Tissues

The four algorithms consistently suggested *RPS15*, *RPS26*, and *RPL13* were the most stable reference genes, whereas *α-tub* and *β-actin* were unsuitable reference genes in different tissues. The RefFinder ranked the overall stability of reference genes for tissues (from highest to lowest) as: *RPS26* > *RPS15* > *RPL13* > *EIF4A* > *UBI* > *AK* > *β-actin* > *α-tub* (Figure 2).

#### 3.3.3. Reared on Different Plants

The ranking of the stability of candidate reference genes in regard to the larvae feeding on different plants was diverse according to the four algorithms. The most stable reference gene was *RPS15* according to the geNorm algorithm and ΔCt method. However, *β-actin* was the most stable reference gene according to BestKeeper. In addition, the lowest M value for the *RPS26*/*RPS15* pair (0.189) calculated by geNorm, indicated they were the most stable transcripts. RefFinder evaluated the stability ranking of eight candidate reference genes from the most to the least stable as *RPS15* > *RPS26* > *α-tub* > *β-actin* > *AK* > *EIF4A* > *UBI* > *RPL13* (Figure 2).

#### 3.3.4. Different Larval Density

BestKeeper and NormFinder suggested that *UBI* and *AK* were the most stable genes in different larval densities, whereas the ΔCt method evaluated *RPS15* as the most stable gene, geNorm identified *RPS15* and *RPS26* as the best pair. *α-tub* was again identified as the least stable reference gene by all four algorithms (Table 1). The RefFinder ranking was: *RPS15* > *RPS26* > *RPL13* > *EIF4A* > *UBI* > *AK* > *β-actin* > *α-tub* (Figure 2).

#### 3.3.5. All Samples

In the final experiment, candidate genes were evaluated to determine the optimal reference gene in all samples. The ΔCt method and NormFinder suggested that *RPS15* and *RPS26* were the two most stable genes. Similarly, the geNorm identified *RPS15* and *RPS26* as the best pair, while BestKeeper ranked *EIF4A* as the most stable gene. Furthermore, *β-actin* was selected as the least stable reference gene by all four algorithms in all samples. The RefFinder ranking was: *RPS15* > *RPS26* > *AK* > *EIF4A* > *UBI* > *RPL13* > *α-tub* > *β-actin* (Figure 2).

### 3.4. Determination of the Optimal Number of Reference Genes for Normalization

**Figure 3:**
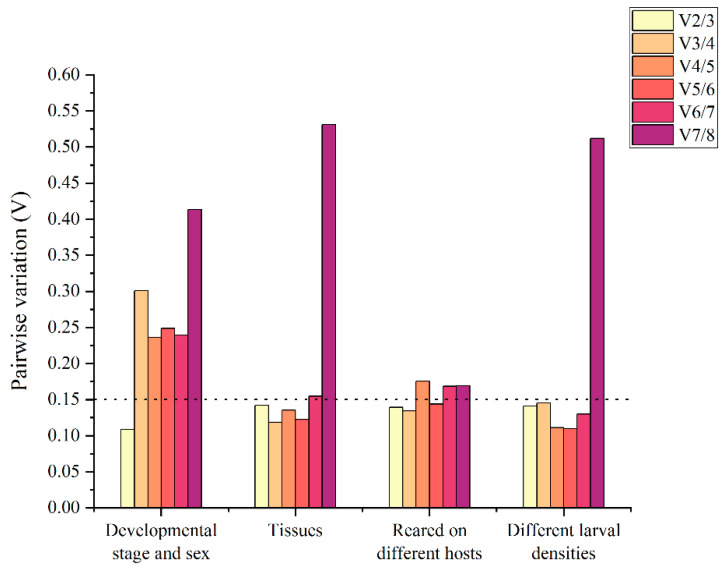
Determination of the optimal number of reference genes according to geNorm. The pairwise variation values below 0.15 indicated that no additional reference gene was needed for the normalization.

Based on the pairwise variation (Vn/Vn+1) of all experimental conditions calculated by geNorm, all experimental conditions showed values below the proposed 0.15 cut-off value at V_2/3_. This indicated that combining the top two reference genes would be sufficient for the normalization of gene expression data (Figure 3).

### 3.5. Validation of Selected Reference Genes in H. cunea

The relative transcript levels of the target gene *HcSP1* under all conditions were normalized to verify the stability of the selected reference genes by the top-ranked genes, the least stable gene, and the combination of stable reference genes, respectively.

**Figure 4:**
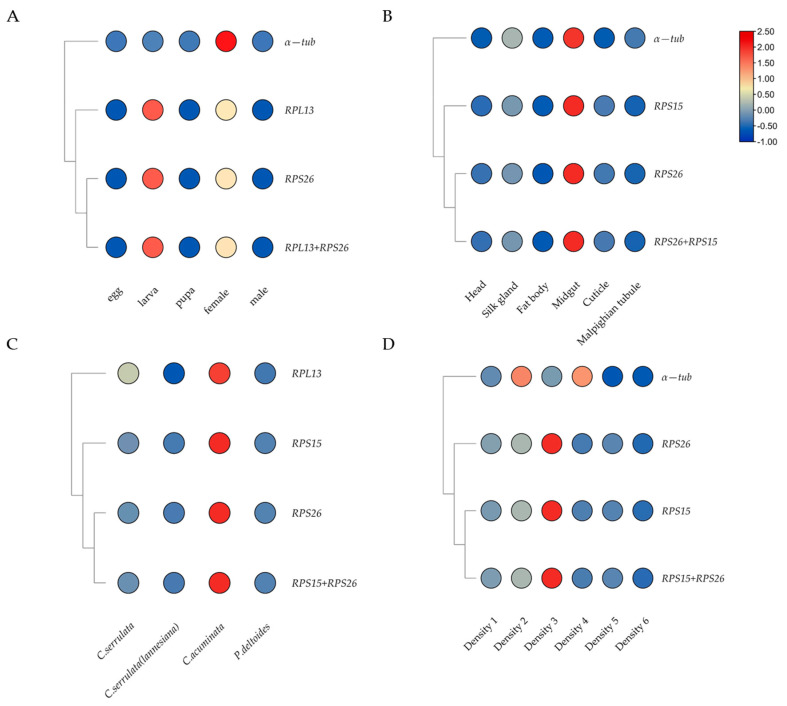
The expression profiles of *HcSP1* under different conditions normalized by selected reference genes. (**A**) Different developmental stages. (**B**) Different tissues. (**C**) Larva feeding on different hosts. (**D**) Different larval densities.

The relative expression level of *HcSP1* was significantly up-regulated in the midgut compared to other tissues, which was normalized by the top-ranked gene (*RPS26* or *RPS15*) (Figure 4). Similar expression-profile changes were obtained by the combination of stable reference genes (*RPS26* + *RPS15*), and there were no significant differences among those normalized by *RPS26* and *RPS15* individually, and *RPS26* + *RPS15*. However, when the normalization by the least stable reference gene (*α-tub*) led to a strong bias in the expression level of *HcSP1* in different tissues, it significantly increased the transcription of *HcSP1* in the midgut and silk glands, and decreased in the head and cuticle. At the developmental stage and different larval densities, the expression levels of *HcSP1* normalized by *RPS26* and *RPS15* individually, or *RPS26* + *RPS15* were different with *α-tub*. While normalized by *α-tub*, the expression of *HcSP1* decreased in the larval stage and density 5 and 6 (12 and 15 larvae per Petri dish) and significantly increased in density 2 and 4. For larvae feeding on different hosts, the expression levels of *HcSP1* were normalized by *RPS26*, *RPS15*, *RPS26* + *RPS15*, and *RPL13*. Although the expression trends were very similar, normalization with the unstable reference gene *RPL13* increased the expression level of *HcSP1* in larvae reared on *C. serrulata* and resulted in larger standard deviation values.

## 4. Discussion

Due to its high sensitivity, rapidity, specificity, and accuracy, the qRT-PCR has become a standard procedure to measure target gene transcript levels and has been widely used for functional gene analysis in insects. However, the assessment of reference genes is an essential procedure to normalize target gene expression and ensure data precision [35,36,37,38,39]. The ideal reference genes must be stably transcribed under any condition, whether it is under different species, varieties, tissues, abiotic and biotic stresses, etc. Unfortunately, when species and experimental conditions vary, selecting the perfect reference gene became problematic. In Lepidoptera insects, such as *Spodoptera litura* (Lepidoptera: Noctuidae) [38]; *Plutella xylostella* (L.) (Lepidoptera, Plutellidae) [40]; *Danaus plexippus* (Lepidoptera, Nymphalidae) [41]; *Sesamia inferens* (Walker) (Lepidoptera, Noctuidae) [39]; *Thitarodes armoricanus* (Lepidoptera, Hepialidae) [42]; *Helicoverpa armigera* (Hübner) (Lepidoptera, Noctuidae) [43], and *Chilo suppressalis* (Walker) (Lepidoptera, Pyralidae) [44]; *Trichoplusia ni* (Lepidoptera: Saturniidae) [45] *Diaphania caesalis* (Lepidoptera, Pyralidae) [46]; *Tuta absoluta* Meyrick (Lepidoptera: Gelechiidae) [47], the stability of reference genes has been well studied, and these studies showed significant differences among different species in different conditions.

*H. cunea* has caused significant economic damage, mainly to forest trees, ornamental plants, and fruit trees. Its molecular physiology and the function of the gene(s) has been actively explored with the publication of genomes [6,11], and recent transcriptomic advances [4,48,49] have opened a window for functional genomics research and interesting gene transcription and regulation studies. In the present study, the stability of reference genes in *H. cunea* was evaluated under different experimental designs by four statistical algorithms (ΔCt, geNorm, NormFinder, and Bestkeeper) to obtain a reliable assessment and avoid selecting co-regulated transcripts. In our results, the most suitable reference gene varied with the conditions. The ranking of genes by geNorm, NormFinder, BestKeeper, and ΔCt algorithms was diverse, probably because different programs have different algorithmic logic [50], and the differences in the scaling systems used by the algorithms can also lead to these variations [35,51]. Although the ranking order varies depending on the analysis program used, the overall trend was similar. Therefore, we used RefFinder, which integrated the four computational programs to comprehensively compare and rank the overall stability of candidate reference genes.

In this study, the geNorm suggested that *RPL13*/*RPS26* was the most stable pair depending on M values in the developmental stages; the ΔCt method and RefFinder were consistent with the geNorm. At the same time, *RPS26* and *RPS15* were identified as optimal reference genes in different tissue types, different larval densities, and larvae reared on different hosts by combining the four algorithms and RefFinder. Besides, it was observed that host plants induced more variations in the Ct values in *H. cunea*. In *Amphitetranychus viennensis*, host plants caused dramatic variations in the Ct values and can cause significant changes in *A. viennensis* at the molecular level [52]. Plants respond to herbivory through various morphological, biochemical, and molecular mechanisms to counter/offset the effects of herbivore attacks. Inhibitors and secondary metabolites produced by host plants may affect the gene expression of herbivores, subsequently affecting their physiology and phenotype [53,54]. In previous studies, under the same rearing conditions, *H. cunea* larvae feeding on different host plants had a significant impact on larval growth and development, feeding behavior, detoxification enzyme activity, and digestive enzymes activity [55,56]. This study suggested that host plants can also cause variations in transcript levels in *H. cunea*.

In the reference gene selection studies, ribosomal proteins exhibited the same stability compared to other types of candidate genes in relation to the abiotic (reared on different hosts and variety of larval densities) and other biotic (developmental stages and multiple tissue types) factors. Other detailed studies have yielded similar results. Ribosomal proteins have been reported as optimal reference genes in several insects. For example, in *Dichelops melacanthus*, *RPS23* exhibited a high level of stability in different genders and tissues as well as *RPL9* for starvation stress and *RPL10* for tissues in *Pagiophloeus tsushimanus* [57,58]. In *Adelphocoris suturalis*, *RPS15* and *RPL32* were identified as the most stable pair for metathoracic scent glands from different developmental stages and gender [59]. In *Bradysia odoriphaga*, *RPS15* was a particularly stable reference gene in different temperature treatments [60]. Our results and previous studies demonstrate that ribosome proteins generally show extremely high stability under diverse conditions in insects. In eukaryotes, ribosome proteins are highly conserved and they are involved in the processes of DNA repair, replication, and transcription; RNA processing, translation, and regulation; protein synthesis; self-translational regulation; and developmental regulation. Therefore, they are used in most eukaryotes for reference genes [61,62].

*β-actin* and *α-tub* were often selected as internal reference genes. For example, actin was the most stable reference gene in development stages, different tissues, and sex of *Diaphania caesalis* [46]. In *C. bowringi* Baly [36], *actin* was used to normalize target gene expressions in the tissues of female adults. In experiments with the *Antheraea pernyi* infected by multicapsid nucleopolyhedrovirus, *actin* and *α-tub* were suitable reference genes for normalizing qRT-PCR data [45]. However, under certain conditions, *β-actin* and *α-tub* were unstable as reference genes. In our research, *β-actin* and *α-tub* showed instability in most conditions (different tissue types, larval densities, and all samples) evaluated by combining four algorithms and RefFinder. Consistent with results for *A. suturalis*, while subjected to several experimental conditions, *β-actin* was particularly inconstant in these conditions [59]. After fungal challenge experiments with *Tribolium castaneum*, *β-actin* and *α-tub* also showed instability for qRT-PCR data normalization [63].

Most studies have shown that combining multiple reference genes rather than a single reference gene can increase the precision of relative quantification [27,64,65]. In this study, when the pairwise variation (Vn/Vn+1) of all experimental conditions was calculated by geNorm, all of the experimental conditions showed values below the proposed 0.15 cut-off value at V_2/3_. This indicates that combining the top two reference genes would be adequate for the normalization of gene expression data.

Furthermore, the accuracy of the reference genes screened by different computational methods needs to be verified, and the *HcSP1* was selected as the target gene. The overall transcriptional pattern of *HcSP1* normalized by the most stable reference gene was similar using a combination of optimal reference genes at different developmental stages, multiple tissue types, different larval densities, and larvae feeding on different host plants. The expression level of *HcSP1* reached a peak at the larval stage when normalized by the top-ranked genes and the combination of stable reference genes, which is consistent with results from *P. xylostella* (L.) [66], *S. litura* [67], and *Mythimna separata* Walker [68]. On the contrary, normalization with *α-tub* showed the highest transcription of *HcSP1* in female adults. Under certain conditions, although expression trends were very similar, normalizing with an unsuitable reference gene affected the relative gene expression and resulted in more significant standard deviations [59]. The expression level of *HcSP1* in the midgut was higher than in other tissues when normalized by the top-ranked genes and combination of stable reference genes, and the same expression pattern was also observed in *P. xylostella* [66,69], *M. separata* [68], and *B. mori* [70]. However, normalization by the least stable reference gene resulted in a strong bias. The transcriptions of *HcSP1* significantly increased in the midgut and silk gland, and decreased in the head and cuticle. Similar results were observed in other conditions. Normalization with the unstable reference genes leads to an inconsistent expression level of *HcSP1*. Consequently, our findings confirm the importance of selecting and validating accurate reference genes for qRT-PCR analysis to avoid the misinterpretation of target gene transcription data.

## 5. Conclusions

In this study, we systematically assessed and validated the transcriptional stability of candidate reference genes in *H. cunea* under multiple experimental conditions. We concluded that ribosomal proteins were the most stable reference genes under different conditions.

*RPS15* and *RPS26* were optimal for different tissues, larval densities, and larvae feeding on different hosts, while *RPS26* and *RPL13* could be used to normalize for different developmental stages and gender. Furthermore, *β-actin* and *α-tub* showed instability in most conditions in this research. The results could lead to a better understanding of the developmental, physiological, and molecular processes of *H. cunea*. In addition, the study provides a comprehensive list of suitable reference genes for analyses and optimal reference genes for other gene function research in *H. cunea*.

## Figures and Tables

**Table 1:** Ranking of the candidate reference genes under different conditions.

Experimental Conditions	Rank	ΔCt		BestKeeper		Normfinder		geNorm	
Developmental stage and sex	1	*RPS26*	1.44	*β-actin*	0.11	*AK*	0.167	*RPS26*	0.205
								*RPL13*	0.205
	2	*RPL13*	1.46	*EIF4A*	0.8	*UBI*	0.563	*—*	*—*
	3	*UBI*	1.49	*UBI*	0.83	*RPS26*	0.653	*RPS15*	0.296
	4	*AK*	1.54	*RPS26*	1.45	*RPL13*	0.704	*AK*	0.756
	5	*RPS15*	1.55	*RPL13*	1.46	*RPS15*	0.865	*UBI*	0.969
	6	*EIF4A*	1.81	*AK*	1.55	*EIF4A*	1.319	*EIF4A*	1.166
	7	*β-actin*	2.2	*RPS15*	1.66	*β-actin*	*1.975*	*β-actin*	1.35
	8	*α-tub*	3.4	*α-tub*	3.56	*α-tub*	3.284	*α-tub*	1.862
Tissues	1	*RPS15*	1.11	*RPL13*	0.33	*RPS26*	0.106	*RPS26*	0.212
								*RPS15*	0.212
	2	*RPS26*	1.13	*RPS26*	0.41	*RPS15*	0.106	*—*	*—*
	3	*RPL13*	1.19	*EIF4A*	0.46	*AK*	0.315	*RPL13*	0.363
	4	*UBI*	1.25	*RPS15*	0.47	*UBI*	0.458	*EIF4A*	0.437
	5	*EIF4A*	1.28	*UBI*	0.76	*RPL13*	0.554	*UBI*	0.553
	6	*AK*	1.3	*AK*	1.01	*EIF4A*	0.764	*AK*	0.637
	7	*β-actin*	1.7	*β-actin*	1.23	*α-tub*	4.242	*β-actin*	0.784
	8	*α-tub*	6.69	*α-tub*	4.11	*β-actin*	4.242	*α-tub*	1.654
Reared on different hosts	1	*RPS15*	1.2	*β-actin*	1.03	*RPS15*	0.094	*RPS26*	0.189
								*RPS15*	0.189
	2	*α-tub*	1.22	*AK*	1.28	*RPS26*	0.153	*—*	*—*
	3	*RPS26*	1.23	*α-tub*	1.73	*α-tub*	0.187	*α-tub*	0.347
	4	*AK*	1.45	*RPS15*	1.82	*AK*	0.263	*AK*	0.601
	5	*β-actin*	1.52	*RPS26*	1.87	*β-actin*	0.471	*β-actin*	0.72
	6	*EIF4A*	1.65	*RPL13*	1.88	*EIF4A*	1.345	*EIF4A*	0.906
	7	*UBI*	1.95	*EIF4A*	2.45	*UBI*	1.808	*UBI*	1.06
	8	*RPL13*	3.85	*UBI*	2.73	*RPL13*	3.813	*RPL13*	1.757
Different larval densities	1	*RPS15*	0.99	*AK*	0.19	*UBI*	0.39	*RPS26*	0.265
								*RPS15*	0.265
	2	*RPS26*	1.02	*RPS15*	0.41	*RPS15*	0.41	*—*	*—*
	3	*EIF4A*	1.03	*RPS26*	0.46	*RPS26*	0.46	*RPL13*	0.384
	4	*RPL13*	1.04	*RPL13*	0.52	*RPL13*	0.52	*EIF4A*	0.501
	5	*β-actin*	1.14	*EIF4A*	0.59	*β-actin*	0.317	*UBI*	0.555
	6	*UBI*	1.19	*UBI*	0.64	*RPL13*	0.347	*β-actin*	0.615
	7	*AK*	1.48	*β-actin*	0.68	*AK*	1.169	*AK*	0.718
	8	*α-tub*	4.15	*α-tub*	4.01	*α-tub*	4.132	*α-tub*	1.568
All samples	1	*RPS15*	2.07	*EIF4A*	1.02	*RPS26*	0.257	*RPS26*	0.514
								*RPS15*	0.514
	2	*RPS26*	2.12	*RPL13*	1.08	*RPS15*	0.257	*—*	*—*
	3	*AK*	2.27	*AK*	1.16	*AK*	0.955	*AK*	0.918
	4	*UBI*	2.31	*UBI*	1.27	*UBI*	1.179	*UBI*	1.071
	5	*EIF4A*	2.47	*RPS15*	1.28	*EIF4A*	1.601	*EIF4A*	1.143
	6	*RPL13*	2.75	*RPS26*	1.52	*RPL13*	1.723	*RPL13*	1.413
	7	*α-tub*	4.38	*α-tub*	4.23	*α-tub*	3.881	*α-tub*	2.188
	8	*β-actin*	5.25	*β-actin*	4.42	*β-actin*	4.942	*β-actin*	2.953

## Data Availability

The data presented in this study are available on request from the corresponding author.

## Supplementary Materials

The following supporting information can be downloaded at https://www.mdpi.com/article/10.3390/insects13010097/s1, Figure S1: Amplification specificity of primers in RT-PCR and qRT-PCR., Table S1: Oligonucleotide primers for candidate qRT-PCR reference genes in *H. cunea*.
