# Supplementary material for: Evaluation of Optimal Reference Genes for qRT-PCR Analysis in Hyphantria cunea (Drury)
Source: Insects. 2022 Jan 14;13(1):97. doi: 10.3390/insects13010097 (PMC8778541; doi:10.3390/insects13010097)
Supplement: Supplementary file 1 [file insects-13-00097-s001.zip › insects-1509914-supplementary.pdf]

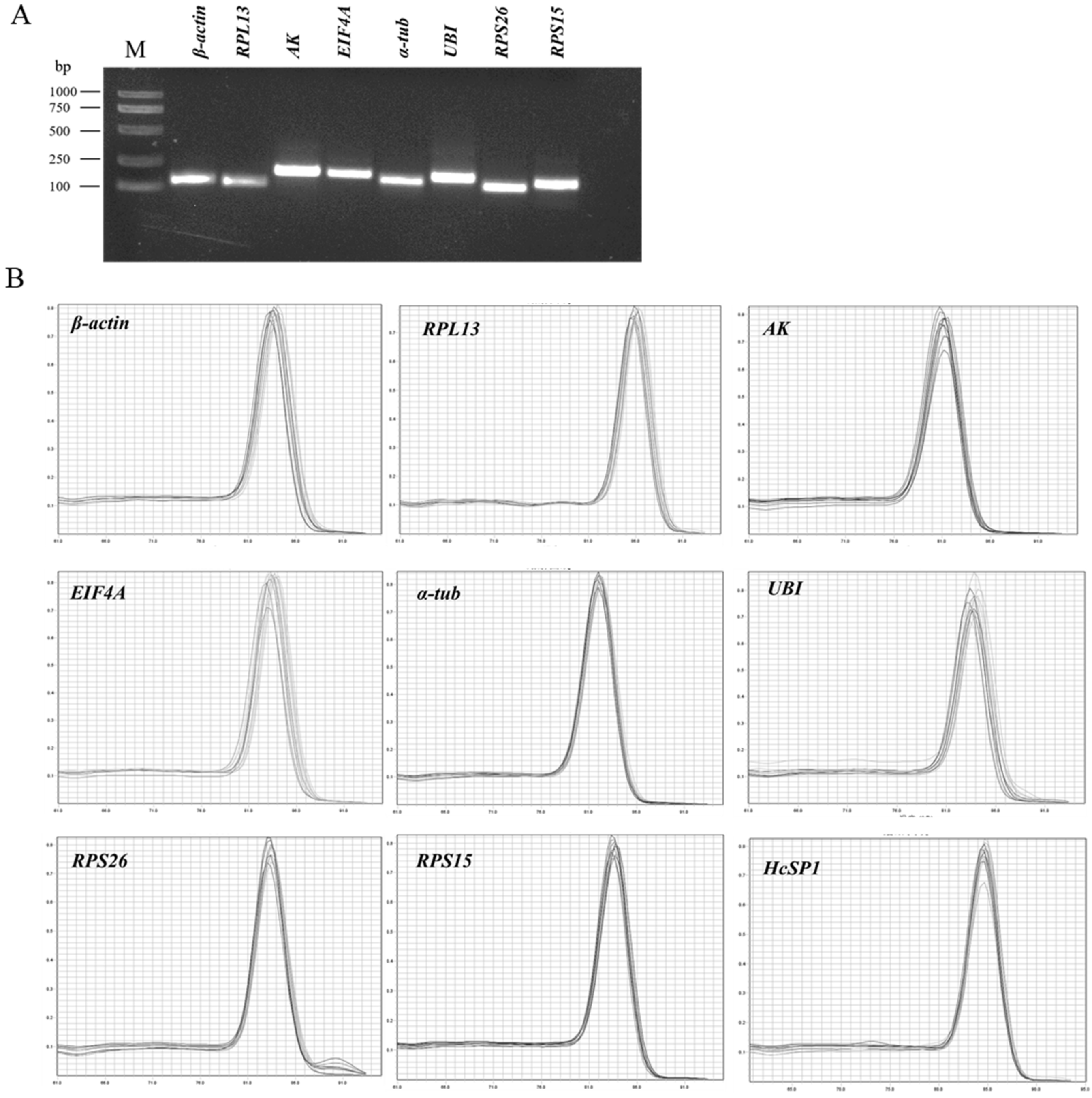

Figure S1. Ampliflcation speciflcity of primers in RT-PCR and qRT-PCR.

A: single amplicon of the expected size for each gene was visualized on a 1 % agarose gel. M, marker. B: single peaks in melt curve analysis.

Table S1. Oligonucleotide primers for candidate qRT-PCR reference genes in *H.cunea*

Gene	GenBank Accession Number	Primer Sequence (5′→3′)	Amplicon Size (bp)	Slope	Efficiency (%)	R^2
β-αctin (*β-αctin*)	OL409114	F：AATGGCTCCGGTATGTGCAA<br>R：ACGAGTCCTTCCGTCCCATA	104	-2.9051	120.91	0.9978
Ribosomal protein L13 (*RPL13*)	OL409115	F：TCCATTAAGGCCGGTTGTCC<br>R：TCCATTAAGGCCGGTTGTCC	142	-2.8027	127.40	0.9741
Argininase kinase (*AK*)	OL409116	F：GTTGAGAGGGAAGCGAGGAT<br>R：GAGTGTGAAAGCGAGCAGAG	130	-3.2262	104.15	0.9997
eukaryotic initiation factor 4A (*EIF4A*)	OL409117	F：TGCGTCGGAAGTGTCGTTTA<br>R：GCAGACGGCTTTTCGAATCC	165	-3.1411	108.14	0.9993
α-tubulin (*α-TUB*)	OL409118	F：TCCATTAAGGCCGGTTGTCC<br>R：AATGCACGGGCTTACAAGGA	92	-3.0535	112.56	0.9996
Polyubiquitin (*UBI*)	OL409119	F：AATCTTCGCCGGCAAACAAC<br>R：CAGAAGGCTCCACTTCCAGG	121	-3.1757	106.48	0.9998
Ribosomal protein S26 (*RPS26*)	OL409120	F：CAGGGACATCAACGAAGCCT<br>R：GGTGCTCTTGGGTGGAGTAC	114	-3.2577	102.75	0.9981
Ribosomal protein S15 (*RPS15*)	OL409121	F：GCGTCACTCTCAAGATCAGC<br>R：ATGGTGCAGATCCTCCAGTC	101	-3.1853	106.03	0.9994
Serine protease1 (*HcSP1*)	MH663425	F：CTGGTGGCATGGATGCTAAG<br>R：TTACAGTCCCTCCACACACC	102	-3.6566	87.70	0.9971
